# Attitudes, knowledge and treatment of low back pain amongst nurses in the Eastern Cape, South Africa

**DOI:** 10.4102/phcfm.v5i1.535

**Published:** 2013-10-24

**Authors:** Liezel Cilliers, Soraya Maart

**Affiliations:** 1 Information Systems Department, University of Fort Hare, South Africa; 2 School of Public Health, University of the Western Cape, South Africa

## Abstract

**Background:**

One of the high-risk professions for the development of musculoskeletal problems is nursing. Studies have reported that there is a high prevalence of low back pain (LBP) amongst South African nurses, but very little is known regarding the prevention and self-treatment principles for LBP in this group.

**Objectives:**

The objective of this study is to evaluate the knowledge, attitudes and beliefs about the prevention and self-treatment principles for LBP amongst nursing staff in Cecilia Makiwane Hospital, Eastern Cape.

**Methods:**

The study population consisted of all qualified nurses employed at the hospital. A cross-sectional survey with a purposive convenience sampling method was used. A questionnaire was designed using literature from established sources. The questionnaire was distributed manually and data obtained were analysed using EPI-INFO4.

**Results:**

The study found that the majority of the participants experienced LBP on a regular basis. The participants could identify the most important physical risk factors associated with the development of LBP, but neglected the psychological risk factors. Action taken after the development of LBP included professional consultations as well as medication and bed rest. The participants identified the different components of a preventative exercise programme but only focused on the physical and not psychological components associated with LBP.

**Conclusions:**

LBP is a serious problem amongst the nurses at the hospital, but no proactive approach is taken in order to address this problem. Policy guidelines and a comprehensive prevention and treatment programme need to be designed and implemented to address this issue.

## Abstrait

**Attitudes, connaissances et traitement des lombalgies chez les infirmiers/-ières dans la province du Cap-Oriental en Afrique du Sud**

**Contexte:**

La profession d'infirmier/-ière fait partie des métiers à risque élevé de développement de troubles musculo-squelettiques. Des études ont indiqué que la prévalence des lombalgies chez les infirmier/-ières d'Afrique du Sud était élevée, mais les principes de prévention et d'auto-traitement des lombalgies dans ce groupe sont très mal connus.

**Objectifs:**

L'objectif de cette étude est d’évaluer les connaissances, attitudes et croyances sur les principes de prévention et d'auto-traitement des lombalgies chez le personnel infirmier à l'Hôpital Cecilia Makiwane, dans la province du Cap-Oriental.

**Méthodes:**

La population de l’étude se composait de tous les infirmiers/-ières qualifiés employés à l'hôpital. Une étude transversale à méthode d’échantillonnage de commodité dirigé a été utilisée. Un questionnaire a été conçu en utilisant des publications de sources reconnues. Le questionnaire a été distribué manuellement et les données obtenues ont été analysées avec EPI-INFO4.

**Résultats:**

L’étude a établi que la majorité des participants souffrait régulièrement de lombalgie. Les participants ont pu identifier les principaux facteurs de risque physique associés au développement de lombalgies, mais ont négligé les facteurs de risque psychologique. Le développement de lombalgies entraînait notamment des consultations professionnelles, la prise de médicaments et l'alitement. Les participants ont identifié les différentes composantes d'un programme d'exercices préventifs, mais se sont uniquement concentrés sur les composantes physiques et non psychologiques liées aux lombalgies.

**Conclusions:**

Les lombalgies représentent un problème grave chez les infirmiers/-ières à l'hôpital, mais aucune approche proactive n'est adoptée pour traiter ce problème. Une politique de directives, une prévention approfondie et un programme de traitement doivent être élaborés et appliqués afin de remédier à ce problème.

## Introduction

Nursing is a high-risk profession for the development of low back pain (LBP).^1^ The prevalence of LBP amongst nursing staff varies according to country. June and Cho^2^ reported that the prevalence of LBP amongst nurses varied from 41% – 75% in European countries, to 40% – 60% in Asian countries and 47% in the United States. Data with regard to the prevalence of LBP in nurses in the sub-Saharan African region is limited, which is disconcerting seeing as the biggest increase in the prevalence of LBP is predicted to be in developing countries.^3, 4, 5^

Non-specific low back pain is defined as low back pain that is not attributable to a recognisable, specific pathology.^6^ As more than 84% of the worldwide population will experience LBP at least once during their lifetime, this disease is now recognised as a major public health problem.^6, 7^ Recent data have shown that in the past decade the proportion of physician visits attributed to LBP has not changed, but the cost of treating LBP has increased substantially.^6^ In about 10 – 15% of patients, acute LBP will develop into chronic LBP. Whilst this percentage is small, this group consumes the most resources through the direct and indirect costs associated with the consequent loss of productivity and earnings.^4, 7^

Of the 126 occupations looked at in a recent American study, nurses ranked the sixth highest with regard to lost working days related to musculoskeletal disorders.^8^ Reasons for this include both extrinsic and intrinsic risk factors that are relevant to this profession. Extrinsic factors include environmental and physical factors, whereas intrinsic factors provide for personal and ergonomic risk factors.^2, 9^ Environmental risk factors include work conditions, the organisational climate and the number of staff members on duty.^2^ A significant relationship has been found between LBP and night shifts as a result of the disturbed sleeping patterns of nurses which can contribute to muscle strain.^2^ Furthermore, mechanical factors such as frequent lifting or transferring of patients and repetitive procedures performed with incorrect or poor body posture have also been identified as risk factors for the development of LBP.^1^ Intrinsic factors include psychosocial predictors such as beliefs about LBP, coping behaviours and psychological distress. Excess weight, low general health status and smoking have also been reported as being possible intrinsic risk factors for the development of LBP.^10, 11^ Karahan et al.^1^ also mentioned that the incidence of LBP seemed to increase in line with the level of stress reported.

Whilst the risk factors for developing LBP amongst nurses have been clearly identified, there are a limited number of studies that evaluate the knowledge of nurses regarding the prevention and self-treatment principles for LBP.^12^ As both clinical evidence and patient preferences should be taken into account when treating this problem, an increased knowledge of intrinsic risk factors for nurses will aid in the better management of the symptom or medical condition.^6^ Understanding the knowledge, attitudes and beliefs about LBP in nurses in developing countries, such as South Africa,

can assist in and contribute to the understanding of the management of LBP on a global scale for all nurses.^5^ The aim of this article is to evaluate the knowledge, attitudes and beliefs surrounding the prevention and self-treatment principles for LBP amongst nursing staff at Cecilia Makiwane Hospital, Eastern Cape. Further objectives included the determination of the prevalence, frequency and duration of LBP amongst the nursing staff at this hospital.

## Research method and design

### Materials

The study population of this study was defined as the 300 nurses employed full-time at Cecilia Makiwane Hospital. Nursing students and nurses on leave at the time of the study were not included as the questionnaire was distributed by hand to the different wards within the hospital. A convenience sampling method was used to identify possible participants. This method was considered suitable as a complete list of nurses employed at the hospital was not available.

The 30 wards in the hospital were allocated to seven departments, namely, the Medical department, Surgical department, Psychiatric department, Paediatric department, Obstetrics and Gynaecology (O + G) department, Emergency Care department (Intensive Care Unit [ICU]) and High Care Unit. In addition, the specialised clinics (Eye, Dermatology and Family Planning) were also included in the study as a separate department.

### Setting

Cecilia Makiwane Hospital, together with Frere Hospital, is part of the East London Hospital Complex (ELHC) in the Eastern Cape. The Eastern Cape Province has consistently recorded the highest number of neonatal deaths in the country over the past five years with the doctor:patient ratio falling 14 times below the national standard reported for South Africa.^13^ It was reported recently that 48% of doctors’ posts and 67% of nursing posts in the province were vacant.^14^

### Design

This research made use of a positivistic, quantitative research methodology. A cross-sectional survey design making use of questionnaires was used to collect data.

Bias in sampling is defined as a systematic error in sampling procedures that leads to a distortion in the results of the study. Sources of bias can include non-response of participants, sampling of volunteers or registered participants only, as well as seasonal and tarmac bias.^15^

In order to minimise bias associated with sampling the data collection tool was pretested and adjusted according to the feedback received. The researcher also visited the wards to encourage non-respondents to complete the questionnaire.

### Procedure

In total, 30 wards in the hospital were identified in which nurses are responsible for patient care. For each ward there is a minimum allocation of two professional nurses and eight nursing assistants. Some specialised units, such as the ICU and the burns unit, have more nurses allocated as they are labour intensive units. The nurse in charge of each ward was asked to identify one nurse with a professional qualification and three nursing assistants to complete the questionnaire. This ratio was chosen to represent the nursing population (one professional nurse to three nursing assistants) of the hospital. This ensured that a wide range of nurses of all categories in different settings and with varied work responsibilities was included in the study. A representative sample size, making use of a confidence level of 95%, a confidence interval of +/- 5 and target population of 300 nurses, was determined to be 169. The questionnaire and a covering letter enclosed in an envelope were distributed by the researcher to the nurses, who were then given two weeks to complete the questionnaire. The researcher visited the wards after the first week to remind the nurses about this task.

The researcher could not find a standardised questionnaire after a search of several data bases (namely, Pubmed, Medline, Free medical journal index, UWC databases and World Health Organization [WHO]). The questionnaire for this study was designed using an informational booklet from the National Institute for Arthritis and Musculoskeletal and Skin Diseases in the United States of America^16^ and The Arthritis Research Campaign booklet^17^ for LBP, as well as other literature found during the literature search.^16, 18, 19^

The questionnaire was designed to provide information related to all the objectives. The questionnaire explored the knowledge, attitude and beliefs of nurses regarding LBP and included the following categories: general information of the participant; past treatment and self-treatment practices; and knowledge on the causes and prevention principles of LBP. It also addresses confounding factors such as gender, age, work place, type of work and duration of career.

### Analysis

Analysis of the data was carried out using EPI-INFO5. Descriptive statistics were used to describe the general characteristics of the sample. The relationship between the prevalence and the knowledge, attitudes and beliefs regarding LBP amongst nurses were evaluated using the Chi-square test. Multivariate logistic regression analysis was used to identify associations between the duration of the participants’ careers and the age of the participant; the prevalence and frequency of LBP; duration of symptoms; and absenteeism. Results of the logistic regression analysis are presented with a 95% confidence interval (CI). A probability level of *p* < 0.05 was accepted as being of statistical significance.

## Results

A total of 150 questionnaires was distributed to nurses working at Cecilia Makiwane Hospital. After a two-week period 109 questionnaires were collected, which represented a 73% return rate The sample comprised 108 women and one man. The average age of the participants was 42.5 years, with (56) 51% of the participants indicating that they were in the 40–49 year age group. Seventy-two per cent (90) of the participants had worked for more than 10 years in the nursing profession.

The majority of the nurses (28; 84%) reported that they had suffered from back pain in the previous week. All the nurses working in the Emergency, O + G and Surgical departments reported that they had suffered from LBP in the past year, with the lowest percentage recorded at 67% in the Psychiatric department.

**FIGURE 1:**
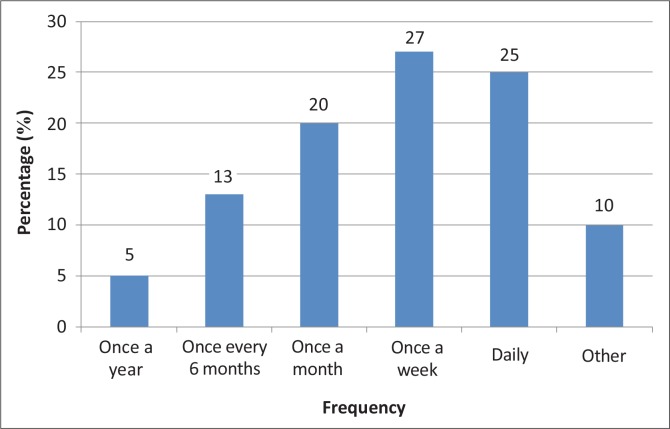
Frequency of low back pain episodes amongst nurses.

Most participants indicated that they had experienced LBP at least once a day (28; 25%) or once a week (30; 27%). Only 5 (5%) reported that they had only one episode a year. Figure 1 shows a breakdown of the frequency of LBP amongst nurses.

Most nurses (85; 78%) reported that their LBP episodes resolved within six weeks. Episodes that lasted for longer than 12 weeks were reported by 21 (19%) of the nurses.

**FIGURE 2:**
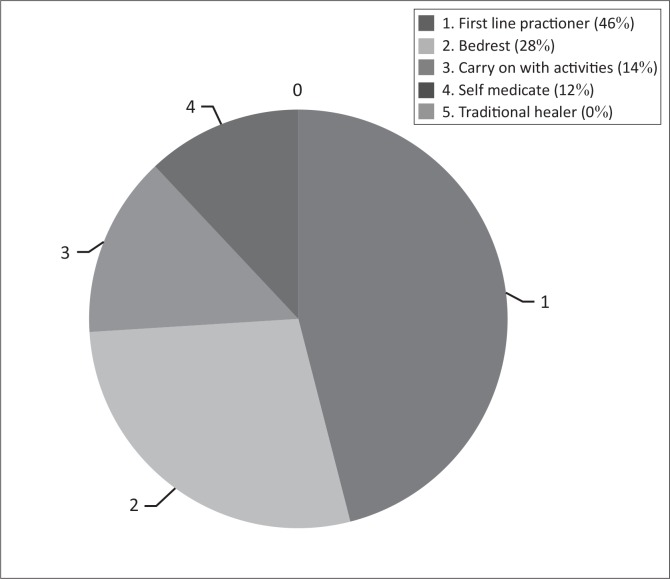
Past treatment practices of participants.

The association between absenteeism and age was of statistical significance (*p* < 0.05). Nurses in the younger than 30 years and 30–40 year age groups were less likely to be absent due to LBP than the 40–50 year age group (Figure 2).

**FIGURE 3:**
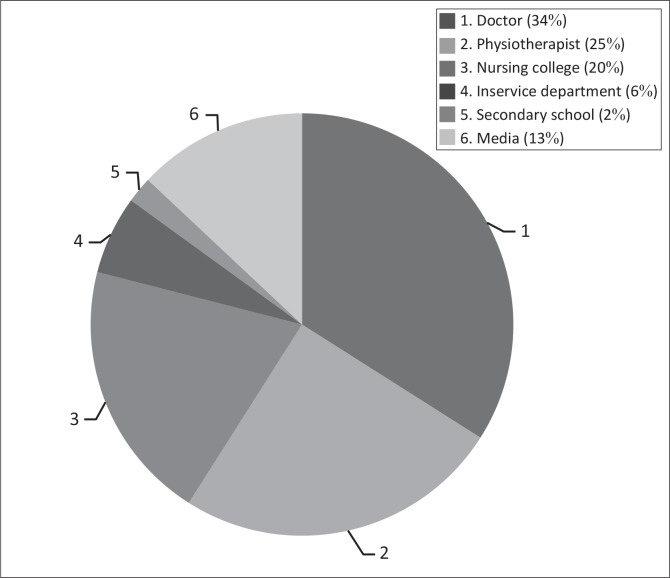
Sources of information about low back pain amongst nurses.

Past treatment practices include the immediate course of action that participants followed after developing LBP as well as which members of the medical team were consulted (Figure 3). The majority of nurses (50; 46%) consulted a first-line practitioner (doctor or physiotherapist). Bed rest was the second most popular treatment option (31; 28%) with only 15 (14%) of nurses opting to carry on with their activities after developing LBP. Twelve per cent (13) of the nurses chose to medicate after the initial LBP incident. No nurses consulted a traditional healer or dietician.

Fifty-four per cent (59) of nurses indicated that they had received some kind of information about LBP in the past. The majority of nurses credited healthcare professionals (doctor 34% and physiotherapist 25%) as the main source of information. Figure 3 provides a more detailed breakdown of the sources.

**FIGURE 4:**
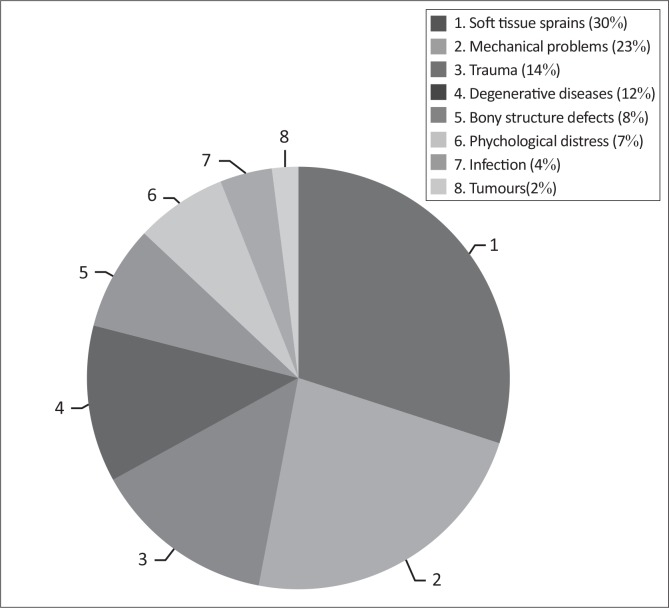
Perceived causes of low back pain amongst nurses.

Only a small percentage of the nurses (8; 7%) indicated that psychological distress could be the cause of LBP. The most popular causes were soft tissue sprains (38; 30%) and mechanical problems (25; 23%), as can be seen in Figure 4.

**FIGURE 5:**
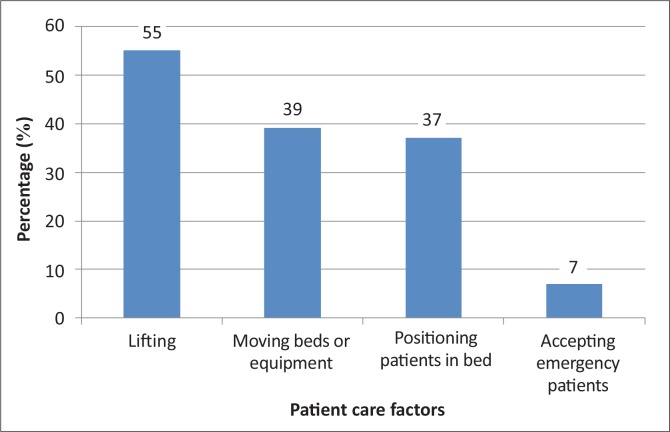
Perceived patient care factors that contribute to low back pain.

Physical or extrinsic factors that were thought to cause LBP included prolonged standing (60; 55%), poor posture (48; 44%) and bending forward (41; 38%). Twenty per cent (22) of the participants considered poor physical fitness to be a factor and slumping was seen as a factor by 17 (16%). The patient care factors that were thought to contribute to LBP included lifting (60; 55%), moving beds or equipment (43; 39%) and positioning patients in bed (40; 37%). Only 8 (7%) of participants considered accepting emergency patients to be a contributing factor (Figure 5).

The intrinsic factors included obesity, age and social factors such as smoking and low educational levels. The participants thought that obesity (78; 72%) and increased age (60; 55%) contributed greatly to the development of LBP. Ten per cent (11) of the participants indicated that they did not believe that any of the social factors given were responsible for the development of LBP.

Psychological factors that contributed to LBP were identified as follows: fatigue (63; 58%), emotional distress (46; 42%) and depression (39; 36%). Fifteen per cent (16) did not believe that any psychological factors could cause or contribute to LBP.

**FIGURE 6:**
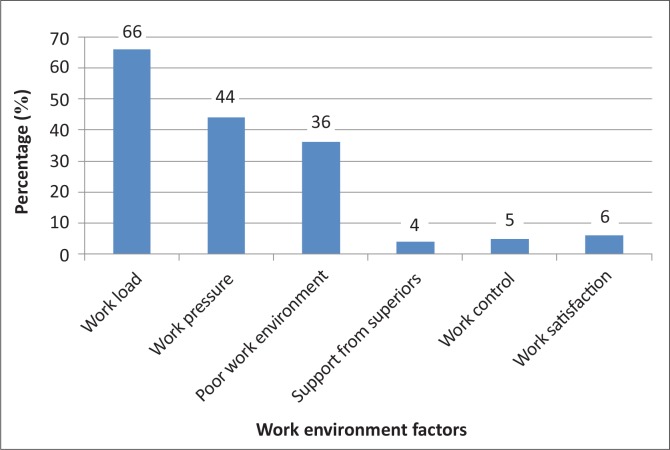
Perceived work environment factors that can contribute to low back pain.

Figure 6 illustrates the work environment factors that were indicated as being responsible for the development of LBP by the participants. These included work load (72; 66%), work pressure (48; 40%) and a poor work environment (39; 36%). Support from superiors, work control and work satisfaction were chosen as being factors by 4 (4%), 5 (5%) and 7 (6%) of the participants respectively.

The best practices for the treatment of LBP were perceived as follows:

- 67% (73) of nurses thought one should consult a doctor.
- 51% (56) preferred bed rest after developing LBP. A period of two to three days was the most popular at 19% (21), followed by a week's rest (17; 16%). Only 8 (7%) of the participants thought it best to carry on with activities when experiencing LBP.
- 39% (43) of the nurses thought that it was best to avoid any activities that could cause more pain when experiencing LBP until the pain is gone.
- 6% (7) indicated they would resume normal activities as soon as possible.

When questioned about what should be included in an LBP prevention programme, the participants indicated that back exercises (54; 50%), weight loss advice (52; 48%) and instructions on how to use lifting equipment (44; 40%) were important topics. Ergonomic principles were only thought to be important by 16 (15%) of the nurses. Psychological aspects such as time management (4; 4%) and relaxation methods (26; 24%) were not regarded as being as important as the physical aspects associated with the development of LBP.

### Ethical considerations

This study was approved by the Research Ethics Committee of the University of the Western Cape as part of a master's study titled ‘Evaluating the knowledge, attitudes and beliefs about the prevention and self-treatment principles for low back pain amongst nursing staff in Cecilia Makiwane Hospital, East London Hospital Complex’. Further permission was also obtained from the Cecilia Makiwane Hospital Management. The respondents received written information explaining the aims of the study and were asked for their consent before partaking in the study. Respondent confidentiality was ensured by making use of a coded system to identity the questionnaires whilst at the same time protecting the identity of the participants. The questionnaires were kept in a locked cabinet with only the researcher having access to them and were then destroyed after the study.

## Trustworthiness

### Reliability

Reliability was ensured through the following:

- Guidelines as set out in the proposal were followed whilst developing, distributing and analysing the questionnaire with the aim of improving standardisation.
- Frequent cross checks were carried out to improve the accountability of data entering.
- A pilot study was conducted to ensure the reproducibility of the questionnaire.
- Good definitions were provided for all variables in order to ensure reproducibility of the questionnaire.
- The questionnaire was translated into isiXhosa, then retranslated into English so as to minimise translation bias.

Questionnaires not returned within the allotted time period were followed up to minimise the ‘healthy worker effect’.^20^ The ‘healthy worker effect’ can create bias when only participants at work are included in the study. Those participants not at work may be absent due to back pain and if special consideration were not given to this in the study the wrong study population would be included which would affect the final results.

### Validity

To ensure the validity of the questionnaire a pilot study was conducted two weeks prior to the main study. The questionnaire was distributed to 10 contract nurses to be tested for user friendliness and clarity. No changes were made to the questionnaire after the pilot study as all questions were found to be clear.

The questionnaire was compiled in English, translated into isiXhosa and then re-translated back into English. Adequately-translated questions were important in this study, as isiXhosa is the first language of most of the study population.

## Discussion

The prevalence of LBP in the current study is 84%. Three departments (Emergency, Surgical and O + G) reported a 100% prevalence. The reasons for the high prevalence in these departments may be because of the unique time constraints and workloads in the department. Nurses are required to respond immediately to emergency situations. The lack of rest and continuous movement during these situations can lead to LBP injuries.

The majority of participants experienced LBP at least once a month with more than half indicating that they had experienced LBP on a daily or weekly basis. Smedley et al.^21^ reported that the risk of recurring LBP increases with both the duration and frequency of previous symptoms. These statistics put the nurses at risk for future injuries and absenteeism from work due to LBP problems.

Seventy eight per cent (85) of the nurses reported that their LBP symptoms had resolved within six weeks. When a nurse is working whilst injured or absent from work because of LBP, it creates a burden on the rest of the staff as they must take on added responsibility.

The general risk factors that were identified as having a potential to contribute to LBP were those to do with the physical aspect of their work. These include the physical condition of the nurse, the physical attributes of the work environment and patient care. The most common perceived causes of LBP identified in the present study were physical factors such as soft tissue sprains, mechanical problems, trauma and degenerative diseases. However, according to Mounce,^12^ less than 25% of all back-pain injuries have an identifiable cause. Of the 25%, only 3% are caused by pathology such as infections, tumours and trauma.^18^

Only a few participants in the present study indicated that LBP could have a psychological cause. If the participants in the study do not understand what the root cause of their LBP is, they cannot reasonably be expected to avoid or manage the pain. They will also not find any benefit in an integrated treatment programme if they do not understand why psychological aspects are included.

The organisational culture of the work unit has been shown to be related to the occurrence of LBP. Participants in this study believe that work load and pressure, but not work control, would contribute to LBP. This is important as the ability to pace oneself when working (i.e. not only the amount or urgency of the work) will help to minimise the risk of developing LBP. Work load, work pressure and a poor environment at work were chosen by participants as being the most relevant factors that contribute to LBP whilst work status, work control, work satisfaction and support from supervisors were not thought to be contributing factors. Management of the hospital also needs to be made aware regarding the risk factors for development of LBP as it causes decreased work efficiency, absenteeism and loss of human resources due to resignations or medical boarding.

Lifting of patients was identified as being the main patient care activity that could cause LBP. These included lifting heavy patients, repetitive lifting and lifting alone. The reasons why nurses tend to injure their backs during transfers include loss of balance (nurse and/or patient), no transfer device, sudden movement and a poor physical work environment. Positioning patients in bed and washing patients were the other activities that were also regarded as high risk. This is supported by Jensen et al.^9^ who found that the frequency of positioning patients in bed also predicted the development of LBP. Only 8 (7%) of the participants identified accepting an emergency patient as being dangerous, which is surprising seeing as all (100%) of the participants had experienced LBP in the Emergency department. According to a study performed by Bongers et al.,^19^ accepting emergency patients may be a risk factor for the development of LBP because of the time pressure entailed. They postulated that more hurried movements, quick accelerations and poor posture are used during busy periods in emergency areas where immediate attention is needed, which will increase the mechanical load on nurses’ backs.

Only 16 (15%) of the participants thought that psychological distress was a risk factor for LBP. This finding is worrying because Smedley et al. found in a previous study that back complaints can be linked to low mood, stress and job dissatisfaction.^21^ The psychological distress causes the patient to be more aware of bodily symptoms such as pain and can increase with the duration of the symptom and the number of specialists seen.^12^ If nurses do not recognise the importance of this contributing factor, they will simply treat the symptoms of the LBP which will cause only temporary relief and not resolve the problem. The other important reason why this factor must be addressed is the development of chronicity amongst LBP sufferers.

Smoking was not regarded as being a risk factor for LBP by any of the participants, even though several studies found that smoking is a consistent risk factor for LBP. The National Institute of Arthritis and Musculoskeletal and Skin Diseases^16^ provide evidence that smoking decreases the absorption of nutrients by the discs in the back. It also slows healing and leads to a prolonged pain experience.

Fifty-four per cent (59) of the participants indicated that they had received some kind of information about LBP. Only a small percentage of the participants reported receiving information from the nursing college curriculum, in-service department of the hospital or mass media. Providing information about LBP has several benefits as it improves beliefs about LBP, self-reported disability and the activities of daily living.

The participants believed that after the development of LBP, activities that cause pain should be avoided, with only a small percentage reporting that they carried on with their normal activities. Almost half (52) of the participants indicated they would avoid all activity until the pain was gone. This belief is in contrast to the National Institute of Arthritis and Musculoskeletal and Skin Diseases^16^ guidelines which advise against bed rest and recommend a gradual return to normal activities. The only indicator for bed rest of no more than four days is initial symptoms of pain radiating down the legs.

There is a body of literature that suggests that an exercise programme can be an effective prevention and treatment modality.^22^ The benefits associated with a general exercise programme include an improved general attitude, decreased depression, reduced stress and muscular tension, as well as a decrease in new back problems, which work together toward to the prevention and/or reduction of LBP.^22^

Most participants recognised that back muscles should be targeted in an exercise programme but only a small percentage of participants indicated that leg and abdominal muscles should be included. The fact that most of the participants did not think that abdominal muscle exercises were important means that they do not have knowledge on the matter. Abdominal muscles provide stability and control to the spine and should therefore be prioritised in LBP programmes.^18^ Studies have shown that there is a greater frequency of LBP amongst patients with poor abdominal muscle function. This is thought to be because the endurance of the back muscles is affected negatively when the abdominal muscles are weak.^22^ In the current study, strengthening and stabilising exercises received little support and endurance training for job dimensions even less. The composition of an exercise programme should include stretching, balancing and general fitness exercises.

It is very important to involve staff when assessing the risks and putting together a back programme for prevention of injury as it has been shown in previous studies that interventions based purely on training about techniques are not effective to reduce LBP. Including staff when planning a back programme will increase ownership of the programme. It will also highlight the perceived and real problems that must be addressed during the programme.

## Limitations

The following limitations are considered for this study. Firstly, the study is cross-sectional and thus gives a weak level of evidence of the association between the measured variables. Secondly, the reliance on the respondents’ self-reporting and recall of events could have led to measurement and recall biases. Lastly, the study used a questionnaire for data-gathering purposes that pre-imposed categories and thus limited the amount of new information that could be produced. However, as a well-established topic in international research literature, it is then assumed that this pre-imposed information will be relevant to the South African context. Further research must be conducted on LBP in nurses to establish the magnitude of the problem in the healthcare sector of South Africa.

## Recommendations

The recommendation from this study include that a comprehensive policy guideline that will address the management of LBP amongst staff must be put in place and made available to staff. In addition a procedure needs to be developed that can provide guidance when incidents concerning LBP occur.

A comprehensive back programme, including physical and psychological components, must be developed in consultation with nursing staff and implemented for staff in the hospital. This programme can be both preventative and rehabilitative in nature.

## Conclusion

The majority of nurses, regardless of where they work at present, experience LBP on a regular basis. In three departments, all the participants indicated that they experienced LBP. More than 60% (67) of the participants experience LBP on a weekly or daily basis. Combined with the absenteeism results of this study it is fair to state that the work performance of the participants is suffering due to LBP.

Even though 54 (half) of the participants indicated that they had not received information about LBP, most of them could identify the most common physical risk factors associated with the development of LBP. The actual perceived causes of LBP included mechanical problems, trauma and sprains of the soft tissue. Psychological risk factors associated with LBP were neglected by the majority of nurses.

Physical factors that were thought to cause LBP included prolonged standing, poor posture and bending forward. The actual movements that were thought to result in LBP, if performed incorrectly, included trunk flexion and extension, but not trunk rotation. Patient care factors associated with the development of LBP included lifting, moving equipment and positioning of patients in bed. Social factors that were thought to contribute to LBP were age and weight. Fifteen per cent (16) of the nurses did not think that LBP could be caused by psychological factors. The rest indicated that fatigue, emotional distress and depression contribute to the development of LBP. Work environment factors included work load, pressure and a poor working environment.

Participants indicated that they preferred to consult a doctor, rest and take medication after the development of LBP. The amount of time the participants wanted to rest was more than clinical guidelines permit. No participants were referred to a dietician for weight-loss advice.

Participants indicated that an exercise programme to prevent LBP should include exercises targeting different muscle groups. The most important muscle groups, abdominal and leg muscles, were, however, left out of the programme. Similarly, endurance and strengthening of muscles were not included in the programme by the participants. The participants indicated that topics that should be covered in a preventative exercise programme include instruction on how to use lifting equipment and weight loss advice, but not time management and ergonomic instruction.

The physical and patient care factors are without doubt the most important contributors to LBP in the nursing profession. It is an integral part of the job that cannot be avoided and must therefore be adjusted to become safe for both the patient and the nurse. It is of equal importance that nurses understand and are aware of the risk factors for LBP. If not, they cannot make provision for safe working and handling practices which will prevent the development of LBP.
